# Serum cytokine profile of neonatal broiler chickens infected with *Salmonella Typhimurium*

**DOI:** 10.3389/fphys.2024.1359722

**Published:** 2024-02-23

**Authors:** Allison Milby-Blackledge, Yuhua Farnell, Dan Zhao, Luc Berghman, Craig Laino, Melissa Muller, J. Allen Byrd, Morgan Farnell

**Affiliations:** ^1^ Texas A&M AgriLife Research, Department of Poultry Science, College Station, TX, United States; ^2^ Millipore Sigma, Saint Louis, MO, United States; ^3^ United States Department of Agriculture, Southern Plains Agricultural Research Service, College Station, TX, United States

**Keywords:** immune response, immunoassay, poultry, serum cytokine, *Salmonella Typhimurium*

## Abstract

The avian immune system responds to *Salmonella* infection by expressing cytokines and chemokines. We hypothesized that the immune status of *Salmonella* Typhimurium (ST) challenged neonatal broilers would differ from the uninfected treatment. The objective of this experiment was to evaluate 12 cytokines. Day of hatch male chicks were randomly allocated into a control or ST challenged group. At day three of age, sterile diluent or 5.0 × 10^8^ CFU of ST was given orally to each chick. Blood was obtained 24 h post challenge and serum separated for later analysis (n = 30 chicks/treatment). Significant (*p* ≤ 0.05) increases in ***pro-inflammatory cytokines***-interleukin-6 (IL-6), IL-16, and IL-21; ***anti-inflammatory cytokines***- IL-10; ***chemokines***-regulated on activation, normal T cell expressed and secreted (RANTES), macrophage inflammatory protein-1β (MIP-1β), and MIP-3α; ***colony stimulating factors***-macrophage colony-stimulating factor (M-CSF); and ***growth factors***-vascular endothelial growth factor (VEGF) were observed in the serum of the challenged chicks when compared to the control. No significant differences were observed in IL-2, interferon gamma (IFNγ), and IFNα. These data indicate the detection of mucosal immune responses in broiler chickens following ST infection. The heightened levels of pro-inflammatory cytokines, chemokines, and colony stimulating factors align with known inflammatory mechanisms, like the influx of immune cells. However, the elevation of IL-10 was unexpected, due to its immunoregulatory properties. Notably, the rise in VEGF levels is compelling, as it suggests the possibility of tissue repair and angiogenesis in ST infected birds.

## 1 Introduction

Salmonellosis is a zoonotic disease caused by *Salmonella* enterica serovars resulting in an estimated 1.4 million cases of foodborne illness and 400 deaths in the United States annually (CDC, 2022). There are more than 2,600 *Salmonella* enterica serovars, but less than 100 of these serovars are known to cause human salmonellosis (USDA-FSIS, 2021). *Salmonella* Typhimurium (ST), *S.* Enteritidis (SE), and *S.* Kentucky (SK) are the three most common isolates found in contaminated poultry products (Van Immerseel et al., 2005; Kumar et al., 2019; Pineda et al., 2021). Poultry infected with paratyphoid *Salmonella* are typically asymptomatic but can continually shed into the environment causing cross-contamination of carcasses and morbidity in humans (USDA-FSIS, 2021).

*Salmonella* are facultative anaerobes that can survive in low oxygen, warm, and humid environments (Parkin et al., 2012; Kurtz et al., 2017). Many *Salmonella* infections occur after ingestion of food or water contaminated with the pathogen (Griffin and McSorely, 2011; Pham and McSorely, 2015; Kurtz et al., 2017). Transmission in poultry is typically via the fecal-oral route (Wigley, 2014; Pham and McSorely, 2015). Once ingested, the organism may adhere to intestinal cells through the pilli or fimbriae where they can colonize the small intestine (Vegad and Katiyar, 2002; Dar et al., 2017). *Salmonella* can then be transported to lymphoid tissues of the gastrointestinal tract and are disseminated throughout the bloodstream (Griffin and McSorely, 2011; Pham and McSorely, 2015). Inflammatory processes will occur and lead to increased expression of cytokines along with an influx of heterophils and monocytes (Wigley, 2014; Swaggerty et al., 2019).

Cytokines are low molecular weight cell signaling proteins that are secreted by immune cells to support and regulate inflammation. They are produced by a variety of cells and play critical roles in inflammation, acute phase protein production, chemotaxis, cell proliferation, and differentiation (Kogut, 2000; Zhong et al., 2019). Pro-inflammatory cytokines are produced by activated macrophages, T helper 1 (Th_1_) cells, and dendritic cells and are involved in the upregulation of immune cells at inflammatory sites (Wigley and Kaiser, 2003; Rothwell et al., 2012; Kaiser and Staheli, 2014). Anti-inflammatory cytokines are a series of immunoregulatory molecules that control the pro-inflammatory responses (Kogut, 2000; Rothwell et al., 2004). Chemotactic cytokines, or chemokines, play a major role in recruiting lymphocytes to the site of inflammation (Mohammed et al., 2007; Sun et al., 2012). They are functionally divided into the CC and CXC superfamilies. These groups are determined by the positioning of cysteine residues (Kirkaldy et al., 2003; Kogut et al., 2005). Colony stimulating factors are a family of growth factors involved in hematopoiesis, while the family known as growth factors are reported to play important roles in cell proliferation, migration, and differentiation during tissue repair and regeneration (Kogut, 2000; Murphy and Weaver, 2017).

The present study examined multiple cytokines during a *Salmonella* infection. We hypothesized that the immune profile of ST challenged chicks would differ from uninfected neonatal broilers. The objective of the current report was to characterize the immune profile of ST infected broilers by evaluating twelve serum cytokines.

## 2 Materials and methods

### 2.1 Experimental design

All experimental procedures were approved by the Texas A&M University Institutional Animal Care and Use Committee (IACUC 2016-0270 and 2019-0171) and the Institutional Biosafety Committee (IBC 2016-112 and 2019-073). Serum samples were used from a previous study (Zhao, 2021). Briefly, day-of-hatch, non-vaccinated male broiler chicks were placed on clean pine shavings in two ABSL-2 floor pens with an environmentally controlled and age-appropriate climate to ensure uniformity. Birds were fed a balanced unmedicated starter diet that met or exceeded industry recommendations for nutrition (Cobb-Vantress, 2018). Upon arrival, chick tray papers were cultured to confirm that the chicks were *Salmonella* negative. After 3 days of acclimation, chicks were orally challenged with 0.5 mL of sterile tryptic soy broth (TSB) or 5.0 × 10^8^ CFU of ST in TSB to ensure a successful challenge of all chicks. The level of colonization of ST-infected chicks was 7.23 ± 0.74 log_10_ CFU/g of cecal contents compared to 0 log_10_ CFU/g of cecal contents observed in the control group. Twenty-four hours post-challenge, blood samples were collected from euthanized birds by cardiac puncture. Blood was collected, kept at room temperature for 2 h, and centrifuged at 2,000 x g for 10 min at 4°C. Serum was separated and transferred into 2 mL aliquots and stored at −80°C for future use. A total of 30 serum samples per treatment were analyzed.

### 2.2 Serum preparation

Serum samples were thawed at room temperature the morning of the experiment. Once unfrozen, serum samples were centrifuged at 10,000 x g for 15 min at 4°C to remove particulates.

### 2.3 Cytokine assays

**FIGURE 1:**
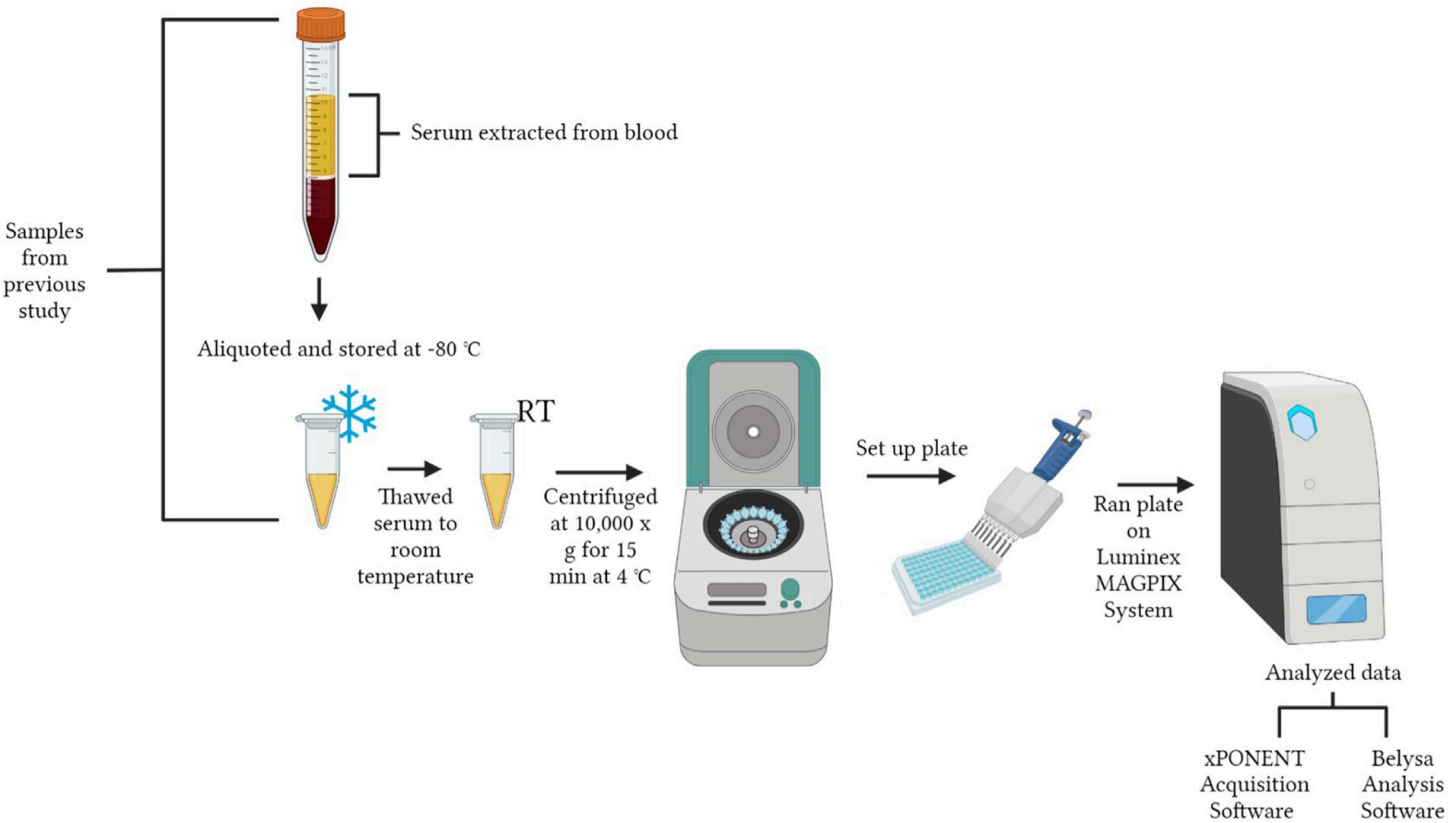
Workflow of experimental design. The figure was created in BioRender.com by Allison Milby-Blackledge.

A workflow of serum preparation and the cytokine procedure is shown in Figure 1. The study was conducted with the Luminex MAGPIX^®^ System (EMD Millipore Corp., Millerica, MA, United States). A MILLIPLEX^®^ Chicken Cytokine/Chemokine Panel (EMD Millipore Corp.) was used to quantify 12 different analytes. Interferon alpha (IFNα), interferon gamma (IFNγ), interleukin 2 (IL-2), interleukin 6 (IL-6), interleukin 10 (IL-10), interleukin 16 (IL-16), interleukin 21 (IL-21), macrophage inflammatory protein-1 beta (MIP-1β), macrophage inflammatory protein-3 alpha (MIP-3α), regulated on activation, normal T cell expressed and secreted (RANTES), macrophage colony-stimulating factor (M-CSF), and vascular endothelial growth factor (VEGF) were evaluated. Detailed definitions of each cytokine are explained in Table 1. The assay was run according to the manufacturer’s instructions with standards, samples and quality controls in duplicate. Premixed antibody immobilized beads, quality controls, wash buffer, and the serum matrix were prepared prior to use. Overnight incubation with shaking at 4°C (16–18 h, 500 rpm) occurred and a handheld magnetic separation block (EMD Millipore Corp.) was used during the plate washing steps.

**TABLE 1:** Analytes observed, where they are produced, their function and type of cytokine response.

Analyte	Produced By	Response	Function
IFNα	Neutrophils, Dendritic Cells, Macrophages	Anti-inflammatory	Anti-viral; MHC I expression
IFNγ	Natural Killer Cells, T cells (Th_1_)	Pro-inflammatory	Macrophage activation; Ig class switching; anti-viral; anti-tumor
IL-2	T cells (Th_1_)	Pro-inflammatory	T cell growth and proliferation; macrophage activation; B cell growth
IL-6	T cells, B cells, Macrophages, Fibroblasts	Pro-inflammatory	Acute phase protein production; B and T cell activation; hematopoiesis
IL-10	Monocytes/Macrophages, T cells, B cells	Anti-inflammatory	Downregulate pro-inflammatory responses; tissue homeostasis
IL-16	B cells, Mast Cells, Eosinophils, Macrophages, Fibroblasts	Pro-inflammatory	Chemotactic activity; MHC II expression; induce inflammation
IL-21	Natural Killer Cells, T cells (Th_1_ or Th_2_)	Pro-inflammatory	Regulate proliferation; immunomodulatory; effector functions of B, T, natural killer, and dendritic cells
M-CSF	Bone Marrow, Fibroblasts, Endothelial Cells	Colony Stimulating Factor	Regulates growth, differentiation, and activation of monocytes/macrophages
MIP-1β/CCL4	Monocytes/Macrophages	Chemokine	Aid in the release of pro-inflammatory cytokines; chemotaxis of neutrophils and lymphocytes
MIP-3α/CCL20	Lymphoid Tissues	Chemokine	Chemotactic activity to epithelial cells surrounding lymphoid tissues
RANTES/CCL5	T cells, Monocytes, Platelets	Chemokine	Recruit leukocytes to inflammatory sites; activation and proliferation of natural killer cells
VEGF	Macrophages, Tumor Cells, Platelets	Growth Factor	Promote growth of new blood vessels

All analyte definitions are comprised from avian immunology literature apart from Janeway’s Immunobiology, ninth Edition.

Kogut, 2000; Wigley and Kaiser, 2003; Min and Lillehoj, 2004; Mohammed et al., 2007; Coble et al., 2011; Yang et al., 2011; Rothwell et al., 2004; Rothwell et al., 2012; Kaiser and Staheli, 2014; Murphy and Weaver, 2017; Al-Khalaifah and Al-Nasser, 2018; Van der Eijk et al., 2019.

### 2.4 Data analysis

Individual microbeads were identified and quantified based on fluorescence signals. Data from the beads were analyzed via Luminex^®^ xPONENT^®^ Acquisition Software (Luminex Corp., Austin, TX, United States) and then exported to the Belysa^™^ Analysis Software (EMD Millipore Corp.) for further examination. A detection target of 50 beads per region was inputted into the software; standards, quality controls, and sample wells with bead counts of less than 36 were excluded. The Belysa^™^ software was used to examine the standard curve. Raw data was transferred from the Belysa^™^ Analysis Software to Microsoft Excel (Microsoft, Redmond, WA, United States) for statistical analysis and removal of outliers.

### 2.5 Statistical analysis

Statistical analysis was performed using JMP Pro 15 (SAS Institute Inc., Cary, NC, United States). Data were analyzed via a Student’s t-test and compiled into Microsoft Excel for further analysis and presented as mean ± the standard error of the mean (SEM). Substantial outliers that exceeded two standard deviations from the mean (SD) were removed. A *p*-value of less than 0.05 was considered statistically significant.

## 3 Results

The levels of cytokines, chemokines, colony-stimulating factors, and growth factors were quantitatively detected. Differences in n may be seen between the control and ST treatments of each analyte due to variation from low bead counts, concentrations below the readable limit, and outliers.

**FIGURE 2:**
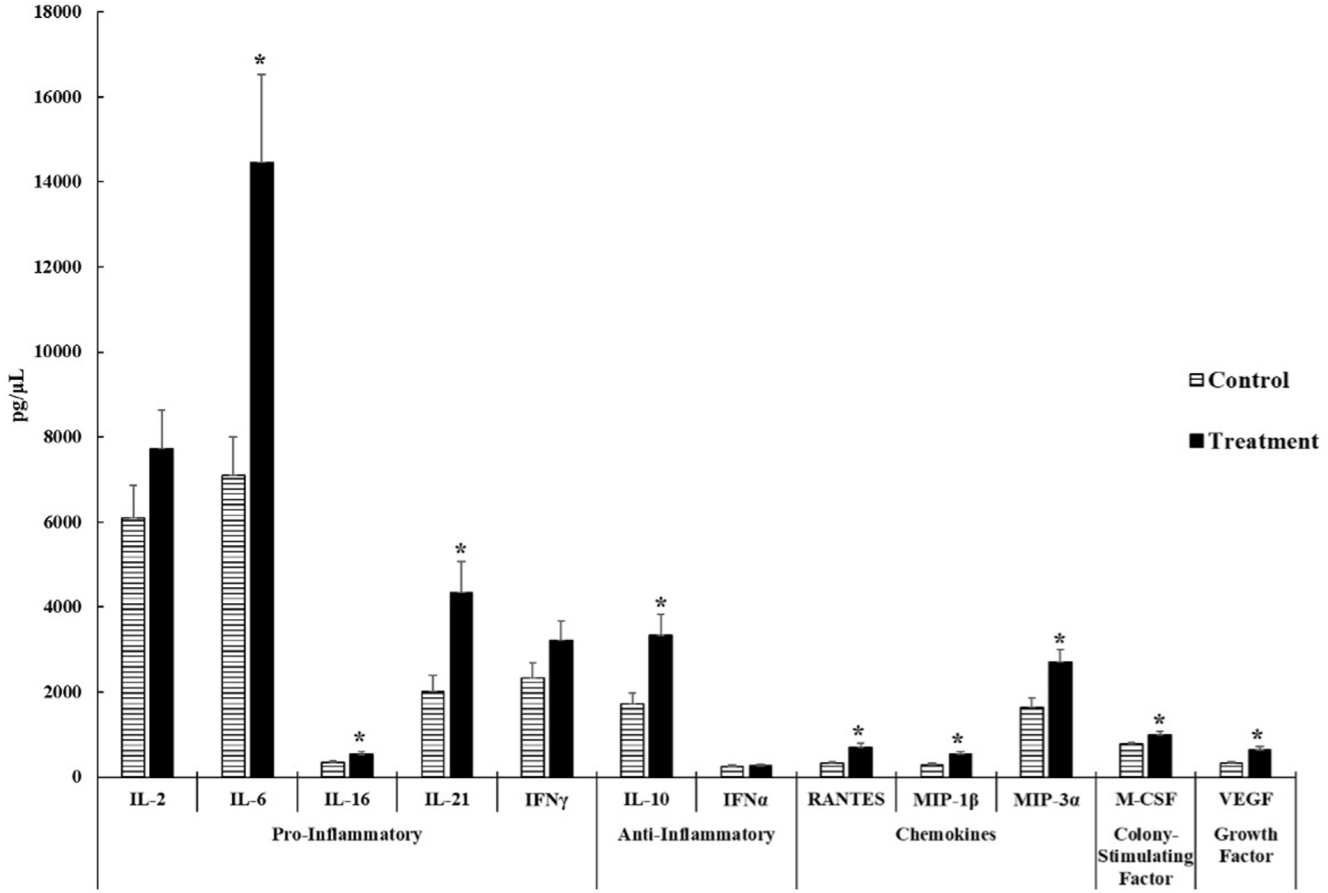
Cytokine and chemokine analysis of the serum of control and *Salmonella* Typhimurium (ST) challenged chicks. Day-old chicks were divided into control and ST treatment groups. Birds in the treatment group were challenged with 5.0 × 10^8^ CFU of ST. The levels of cytokines, chemokines, growth factors, and colony-stimulating factors were analyzed. The target sample size (n) was 30 samples. However, n differs between control and ST treatments of each analyte due to variation from low bead counts, concentrations that were below the readable limit, as well as outliers causing the actual n to range from 23 to 27 samples for the control group and 26 to 29 samples for the ST treatment group. An asterisk (*) indicates concentrations that are considered significant differences at *p* ≤ 0.05 compared to the control group.

As shown in Figure 2, we observed that ST-challenged neonatal broilers after 24 h post-infection had significant increases in ***pro-inflammatory cytokines***- IL-6 (*p* = 0.0025), IL-16 (*p* = 0.0196), and IL-21 (*p* = 0.0066). No significant differences were observed in IL-2 (*p* = 0.1778) and IFNγ (*p* = 0.1316). It is interesting to note that IL-6 displayed a 2.04 fold increase in the ST treatment compared to the control.

The concentration of ***anti-inflammatory cytokine***- IL-10 (*p* = 0.0047) was significantly higher in the ST treatment than in the control. No significant differences were observed on IFNα (*p* = 0.5044) concentrations in both groups. Both cytokines in this group were above the minimum detectable concentrations set forth by the manufacturer.

All ***chemokines*** tested in this experiment: RANTES (*p* = 0.0002), MIP-1β (*p* = 0.0019), and MIP-3α (*p* = 0.0050), exhibited significant increases in the ST group compared to the control group.

The mean concentration of the ***colony stimulating factor*-** M-CSF in the ST treatment was significantly higher than the control group (*p* = 0.0239) with a 1.26 fold increase in the ST chicks compared to control chicks.

The mean concentration of the ***growth factor***- VEGF (*p* = 0.0022) was significantly increased in ST-infected chicks compared to the control. There were 24 control samples and 29 ST-infected samples that were viable.

## 4 Discussion

Cytokines represent a diverse array of secreted proteins that serve as vital communication tools among all cells throughout the body (Eckmann and Kagnoff, 2001). Data is limited when it comes to systematic protein expression in poultry. Protein concentration although generally more conserved than mRNA levels across species, is not widely used to show cytokine levels in poultry (Jiang, et al., 2023). This study was performed to better understand the role the immune system has on combatting ST infection in chickens, herein we report the significant upregulation of protein expression in nine out of the twelve serum cytokines analyzed in ST-infected chicks.

In poultry *Salmonella* infections, intestinal colonization can cause inflammation, specifically to the distal ileum and ceca of birds (Broz et al., 2012; Dar et al., 2017). This can cause rapid expression of pro-inflammatory cytokines in newly hatched chicks (Withanage et al., 2004). Interleukin 6 is a common pleiotropic cytokine associated with *Salmonella* infection (Kaiser et al., 2000). It is a cytokine that stimulates acute phase protein production, inflammation, B cell proliferation and differentiation (Wigley and Kaiser, 2003; Nishimichi et al., 2006; Al-Khalaifah and Al-Nasser, 2018). Many studies performed on mammalian species after *Salmonella* infection or LPS challenge have been evaluated (Franchi, 2011; Abos et al., 2016; Suksungworn et al., 2020; Huang, 2021). Previous studies on mice support our findings of the significant increases in IL-6 protein concentrations following ST infection (Mathur et al., 2012; Febriza et al., 2020). However, many experiments in poultry focus on mRNA levels (Shebl et al., 2010). For example, our study differs from Pineda et al.‘s study, where no significant differences in IL-6 or IFNγ mRNA expression were observed in the broiler cecal tonsils or liver when inoculated with SK, ST, and SE (Pineda et al., 2021). However, our data correlates to Kaiser and associates *in vitro* study, when specifically looking at IL-6 (Kaiser et al., 2000). This study focused on primary chick kidney cells (CKC) to determine the levels of pro-inflammatory cytokines against ST and SE. Interleukin-6 mRNA levels were significantly increased in the ST-infected CKC compared to controls (Kaiser et al., 2000). In the current study, no significant differences were observed in IL-2 which is responsible for T-cell proliferation and activation of macrophages or the antiviral type II interferon, IFNγ (Wigley and Kaiser, 2003). Although parameters were not the same (2 h of contact time with the cells instead of 24 h challenge to the chick), our findings contradict what is found in the literature, as mRNA expression of IFNγ and IL-2 were significantly downregulated nearly fivefold when exposed to ST compared to the control (Kaiser et al., 2000). Interleukin 16 is generated by B cells, epithelial cells, macrophages, mast cells, fibroblasts, and eosinophils. It is considered an inflammatory cytokine in chickens that induces lymphocyte chemotaxis (Min and Lillehoj, 2004; Kaiser and Staheli, 2014). Cytokines have been reported to be expressed in the serum following a *Salmonella enterica* challenge (Song et al., 2020). Our data not only correlates to this statement but also is similar to Swaggerty et al.’s experiment, where the addition of antioxidants in breeder hen diets were used to protect against SE in the progeny*.* In Swaggerty et al.’s study, cytokine and chemokine production was measured in the serum using the MILLIPLEX^®^ Chicken Cytokine/Chemokine Panel and analyzed using the Luminex 200 xMAP Technology. Chicks from the control fed hens had increased levels of IL-16 in the serum (Swaggerty et al., 2023). Since chicks in the protected biofactors and antioxidant (P(BF + AO)) feed additive-fed hen group had a 32.6 percent reduction of SE, the increase in IL-16 levels could be because the immune system is having to work harder to fight off infection which could also be linked to why we are seeing an increase in our ST group (Swaggerty et al., 2023). Interleukin 21 is a cytokine of the cell mediated immune system with immunomodulatory properties. It regulates proliferation, differentiation, and effector functions on T cells, B cells, and natural killer cells (Murphy and Weaver, 2017). In the present study, increases (*p* = 0.0066) of IL-21 concentrations were observed in the ST treatment, indicating the involvement of immune cells. Although interesting, the body of literature for avian IL-21 is limited to a characterization paper (Rothwell et al., 2012) and the effect that *Mycoplasma synoviae* and lentogenic Newcastle disease virus (NDV) coinfection have on gene expression of chick embryos where it was concluded that IL-21 mRNA expression of the liver and spleen were significantly downregulated and the mRNA expression of the thymus was significantly upregulated in NDV-infected embryos (Bolha et al., 2013).

Anti-inflammatory cytokines act as immunoregulatory molecules that return the immune system to baseline or inhibit the Th_1_ response (Opal and DePalo, 2000; Kogut and Arsenault, 2017). Interleukin 10 is primarily produced by T cells, B cells, and monocytes/M2 macrophages (Chuang et al., 2016; Murphy and Weaver, 2017). Much of our understanding regarding the role of IL-10 in infectious disease originates from observations in the murine model. It is generally recognized that decreased IL-10 levels can promote resistance to primary infection, while elevated levels can increase susceptibility to intracellular pathogens (Rothwell et al., 2004). Although some poultry studies demonstrate reduced IL-10 gene expression, post-ST or SE challenge (Fasina et al., 2008; Redmond et al., 2009; Crhanova et al., 2011), our findings correlate to previous research conducted in ST-infected mice where increased IL-10 production results are indicative of B cells and T cells promoting and developing immune tolerance induced by ST (Salazar et al., 2017).

Macrophages can produce a small heparin-binding class (molecules that bind growth factors and promote tissue repair) of cytokine that acts as a chemoattractant to bring leukocytes to infected tissues, better known as chemokines (Martino et al., 2012). The observed outcomes of the current study demonstrated significant increases in MIP-1β (CCL4), MIP-3α (CCL20), and RANTES (CCL5), which suggest the movement of immune cells toward areas of inflammation (Hughes and Nibbs, 2018). Macrophage inflammatory protein-1 beta is an inflammatory chemokine secreted by chicken monocytes/macrophages that induces a pro-inflammatory response and chemotactic migration of heterophils and lymphocytes (Yang et al., 2011; Van der Eijk et al., 2019). A previous investigation performed by Kogut, He, and Kaiser that centered on examining the activation of chicken heterophils triggered by lipopolysaccharide (LPS), showcased the generation of CXC and CC chemokines, notably MIP-1β, in response to infection (Kogut et al., 2005). Our current findings are consistent with the literature, implying that these outcomes indicate the ability of chemokines to guide heterophil recruitment to the inflammation site (Kogut et al., 2005). Macrophage inflammatory protein-3 alpha is a chemokine that recruits dendritic cells during an inflammatory response (Aziz et al., 2016). It is produced by activated epithelial cells and attracts T cells to the site of inflammation (Akahoshi et al., 2003). Reports of MIP-3α in poultry *Salmonella* infection are limited. Recent studies of MIP-3α expression are mainly found in human medicine (Aziz et al., 2016) However, a study performed by Withanage and others, showed increased expression of the MIP family chemokines in the ileum and cecal tonsils of ST infected birds. Although this is not specific to MIP-3α, their results correlate to what is seen in the current study and they speculated that the MIP family chemokines are involved in the recruitment of T cells to the intestines and protective immunity to the host (Withanage et al., 2005). The chemokine RANTES, acts as a pro-inflammatory chemokine, attracts T cells and basophils to the site of inflammation, and induces natural killer cell proliferation and activation (Maghazachi et al., 1996; Coble et al., 2011). Coble and others measured a significant increase in RANTES mRNA levels in broilers infected with *Salmonella* compared to hens (Coble et al., 2011). Dorner and associates found that when looking at the initial expression of all of these chemokines combined in the murine immune system, the role of chemoattractants MIP-1β and RANTES paired with MIP-1α amount to coactivation of macrophages; and when these chemokines are then paired with IFNγ, they act as cell mediated cytokines used by the natural killer cells to bridge components of the Th_1_ response (Dorner et al., 2002).

Colony stimulating factors are a family of growth factors involved in the development, proliferation, and survival of hematopoietic cells (Kogut, 2000). In this experiment, M-CSF is the colony-stimulating factor of interest, which is derived from bone marrow fibroblasts and endothelial cells. Macrophage-colony stimulating factor is a growth factor that regulates the development, proliferation, and differentiation of macrophages (Kogut, 2000; Al-Khalaifah and Al-Nasser, 2018). Previous research has shown that *Salmonella* can cause impairment to M-CSF-induced macrophage recruitment and decreases levels of M-CSF secreted by epithelial cells in mice (Zhang et al., 2014). However, M-CSF has not been widely observed in poultry *Salmonella* infections and data to compare to this study are limited. Other researchers have found M-CSF-like activity in the chicken embryo specifically the egg yolk, amniotic fluid, and chorioallantoic fluid during development (Shao et al., 1994; Shao et al., 1996). Levels in these studies were downregulated after incubation of the egg. These results contradict what is viewed in the current experiment where M-CSF was significantly upregulated in ST infected broilers compared to control broilers. In another study conducted by Sakurai et al., the administration of M-CSF was found to control antigen-specific immune responses due to increases in B cells, natural killer cells, and the activation of murine macrophages for tumor killing (Sakurai et al., 2008). Our results in the current study showed that M-CSF could be an analyte of interest for further research on *Salmonella* infection of poultry, because it could be involved in regulating macrophage and monocyte populations or myeloid cells (Ushach and Zlotnik, 2016).

Growth factors are biologically activated molecules that are secreted in response to cell proliferation, migration, and differentiation during tissue repair and regeneration (Stone et al., 2022). Vascular endothelial growth factor is specifically involved in promoting endochondral ossification and angiogenesis (Zhang et al., 2013; Murphy and Weaver, 2017). Vascular endothelial growth factor is not a typical cytokine of interest for *Salmonella* infection. Previous research uses VEGF for exploring avian diseases like tibial dyschondroplasia (TD; Zhang et al., 2013; Huang et al., 2017), tumor angiogenesis (Duffy et al., 2013), and increasing angiogenesis in chicken embryo membranes (Fernandez and Bonkovsky, 2003). In the current study, we saw significant increases of this growth factor in ST infected broiler chicks compared to the control. We are unsure why VEGF was upregulated in *Salmonella* infected poultry, however, it is plausible that it could be linked to possible tissue repair and blood vessel growth during ST infection.

Our research characterized the immune profile of ST infected neonatal broilers. As we know, immune cells are an important part of the avian lymphatic system, they move systemically through the blood looking for foreign invaders, or they can reside and function in lymphoid tissues (Farber, 2021). Inflammation is a complex process that occurs in response to foreign bodies. It recruits cells from the blood and lymph to infected tissues and produces cytokines (French et al., 2020). After testing serum cytokine levels, these data suggest that the anti-inflammatory properties of IL-10 and the pivotal role IL-6 has in T cell and B cell mediated immunity, could be associated with the switch from cell mediated immunity to humoral immunity (Tan and Coussens, 2007; Choy and Rose-John, 2017; Huang, 2021). Increased concentrations of IL-10 could also be linked to developing tolerance to ST colonization by preventing damage to the host without affecting pathogen numbers (Opal and DePalo, 2000; Dorner et al., 2002; Tan and Coussens, 2007; Kogut and Arsenault, 2017). Furthermore, the increase in VEGF could potentially be linked to promoting restoration of damaged tissues and angiogenesis in ST infected birds based on previous research performed in TD (Zhang et al., 2013; Huang et al., 2017). Although 24 h post-infection is typically viewed as premature for the onset of humoral immunity, elevated IL-6, IL-10, and chemokine levels are compelling and we believe future trials will allow us to further examine these cytokines by using antibody titers to determine B cell development and immune reactions occurring in the body. We also believe that further investigation into cell populations and their involvement with cytokine expression could support the current data that is being presented.

## Data Availability

The raw data supporting the conclusion of this article will be made available by the authors, without undue reservation.
